# Intracortical Causal Information Flow of Oscillatory Activity (Effective Connectivity) at the Sleep Onset Transition

**DOI:** 10.3389/fnins.2018.00912

**Published:** 2018-12-04

**Authors:** Antonio Fernandez Guerrero, Peter Achermann

**Affiliations:** ^1^Institute of Pharmacology and Toxicology, University of Zurich, Zurich, Switzerland; ^2^Neuroscience Center Zurich, University of Zurich and ETH Zurich, Zurich, Switzerland; ^3^The KEY Institute for Brain-Mind Research, Department of Psychiatry, Psychotherapy and Sychosomatics, University Hospital of Psychiatry, Zurich, Switzerland; ^4^Zurich Center for Interdisciplinary Sleep Research, University of Zurich, Zurich, Switzerland

**Keywords:** effective connectivity, default mode network, central executive network, delta activity, sigma activity, sleep deprivation

## Abstract

We investigated the sleep onset transition in humans from an effective connectivity perspective in a baseline condition (approx. 16 h of wakefulness) and after sleep deprivation (40 h of sustained wakefulness). Using EEG recordings (27 derivations), source localization (LORETA) allowed us to reconstruct the underlying patterns of neuronal activity in various brain regions, e.g., the default mode network (DMN), dorsolateral prefrontal cortex and hippocampus, which were defined as regions of interest (ROI). We applied isolated effective coherence (iCOH) to assess effective connectivity patterns at the sleep onset transition [2 min prior to and 10 min after sleep onset (first occurrence of stage 2)]. ICOH reveals directionality aspects and resolves the spectral characteristics of information flow in a given network of ROIs. We observed an anterior-posterior decoupling of the DMN, and moreover, a prominent role of the posterior cingulate cortex guiding the process of the sleep onset transition, particularly, by transmitting information in the low frequency range (delta and theta bands) to other nodes of DMN (including the hippocampus). In addition, the midcingulate cortex appeared as a major cortical relay station for spindle synchronization (originating from the thalamus; sigma activity). The inclusion of hippocampus indicated that this region might be functionally involved in sigma synchronization observed in the cortex after sleep onset. Furthermore, under conditions of increased homeostatic pressure, we hypothesize that an anterior-posterior decoupling of the DMN occurred at a faster rate compared to baseline overall indicating weakened connectivity strength within the DMN. Finally, we also demonstrated that cortico-cortical spindle synchronization was less effective after sleep deprivation than in baseline, thus, reflecting the reduction of spindles under increased sleep pressure.

## Introduction

Falling asleep is a process characterized by the relative disengagement from the external environment and a loss of consciousness, yet, little is known about its neurophysiological basis (Ogilvie, 2001; Marzano et al., 2013). Given that conscious awareness is attributed to the capability to integrate activity originating from diverse brain regions (particularly cortico-cortical and cortico-thalamic interactions), a suitable approach to examine how this process unfolds is by applying connectivity methods which are able to describe how distinct brain oscillations transmit information between brain regions (Massimini et al., 2005; Esser et al., 2009; Chow et al., 2013; Tononi et al., 2016). With a seed-based approach or by independent component analyses (ICA), several brain networks were identified during rest (resting state networks, RSNs), with nodes clustered together according to a shared pattern of temporal correlations (Park and Friston, 2013; Amico et al., 2014). Two prominent networks stand out, the default mode network (DMN, thought to be involved in mind-wandering, creativity and emotional processing) and the central executive network (CEN, involved in cognition, planning and working memory), generally being anti-correlated to the DMN (Buckner et al., 2008; Douw et al., 2014; Salone et al., 2016). This study aims to investigate connectivity changes paralleling the sleep onset (SOn) transition, on a selection of nodes that combines these two principal networks, which have been previously related with consciousness and sleep (Sämann et al., 2011; Heine et al., 2012; Uehara et al., 2014).

Identifying potential interactions arising in a network of connected brain regions is one of the most important and relevant problems in neuroscience (He and Evans, 2010; Park and Friston, 2013). In this context, connectivity can be addressed in three major ways by investigating structural, functional, and effective connectivity (Sporns and Betzel, 2016; Stam et al., 2016). Briefly summarized, structural connectivity deals with the topology of white matter tracts physically linking different brain areas, functional connectivity reflects temporal correlations between regions, and effective connectivity completes and corrects the deficiencies of functional connectivity, by pruning potential indirect connections and by adding causal and directionality aspects (Friston, 2011; Valdes-Sosa et al., 2011; Stam et al., 2016). The use of the EEG, as in this study, and in contrast to fMRI research (typically limited to the mere predominant directionality of slow components due to the low temporal resolution of fMRI), allows for an additional spectral representation of effective connectivity coupling due to its much higher characteristic sampling rate (Pascual-Marqui et al., 2011). In this paper, we will focus on effective connectivity to study the SOn transition from a connectivity perspective including directionality.

Effective connectivity methods are mathematical tools created to best characterize the influence that one neuronal group is impinging on another one in the context of neuronal networks (in principle, composed of an arbitrary number of nodes) (Friston, 2011; Liu and Aviyente, 2012). Pioneers in the study of causal relationships among a set of signals were Akaike (with a method called Noise Contribution Ratio) and Granger (with a causality method named after him), laying the analytical foundation for this field, which has advanced substantially since then, yet, continuing to be under development (Akaike, 1968; Granger, 1969). In general, effective connectivity methods can be formulated either in the time or frequency domain and can have either linear or non-linear characteristics in the underlying equations (Geweke, 1982; Bakhshayesh et al., 2014; Khadem and Hossein-Zadeh, 2014). Some well-known effective connectivity methods are: Granger Causality (GC), Partial Directed Coherence (PDC), Transfer Entropy (TE), Phase Slope Index (PSI) or Dynamic Causal Modeling (DCM) (Barnett et al., 2009; Silfverhuth et al., 2012; Ewald et al., 2013; Friston et al., 2014).

In order to assess effective connectivity changes characterizing the SOn transition, we applied isolated effective coherence (iCOH) (Pascual-Marqui et al., 2014a). Intuitively, this method provides a normalized measure (between 0 and 1) of the coupling strength between a pair of nodes in the frequency domain, which is sensitive to the directionality of the interaction by index permutation (i.e., *A* directs *B*, versus the opposite, which can vary remarkably, a distinction neglected by functional connectivity methods) (Kralemann et al., 2014). By mathematical construction, iCOH prunes away any indirect path linking two particular brain areas, which could distort the results in a major way (especially for complex networks). For example, if node *A* directs *B*, and *B* directs *C*, but there is no direct connection between *A* and *C* (existing only indirectly), then, iCOH results preserve the actual causal order, whereas other techniques (e.g., GC or Directed Transfer Function) would tend to incorrectly indicate a direct connection between *A* and *C*, biasing the causal structure of a network (Baccala and Sameshima, 2001; Faes et al., 2010). In addition, the frequency representation of iCOH allows to study the relevant information contained in its spectral characteristics and can be used to deduce which frequency bands (indicated by either absolute or local maxima) are preferentially involved in the information transfer between a set of brain regions (Pascual-Marqui et al., 2014a).

## Materials and Methods

### Data Description and Preprocessing

The EEG data analyzed in this study are from recordings in eight healthy young right-handed men of a previous study (Finelli et al., 2000, 2001a,b). The EEG signals were sampled at 128 Hz [band-pass filter: 0.16–30 Hz; for additional details see (Finelli et al., 2001b)]. The sleep stages were scored for 20-s epochs (C3A2 derivation) according to Rechtschaffen and Kales (1968). Artifacts were identified as described in Finelli et al. (2001b). Additionally, 4-s epochs containing artifacts were replaced by the preceding epoch free of artifacts (12-s epochs, i.e., three consecutive 4-s epochs were analyzed). The total number of recording channels was 27, placed evenly on the scalp according to the international 10/20 system. Although a technical reference (placed 5% rostral to Cz) was originally used in the recordings, in the subsequent processing, all EEG data were re-referenced to the average reference. EEG recordings were performed during a baseline night (bedtime 23:00–7:00 hours), and recovery sleep (23:00–11:00 hours) following 40 h of sustained wakefulness (within-subject design). The local ethics committee approved the study protocol and written informed consent was obtained from the participants prior to the study. For further details on participants (inclusion, exclusion criteria; protocol) and recordings see (Finelli et al., 2000, 2001b). Effective connectivity data were computed by means of iCOH (implemented in eLORETA) in the following frequency bands: delta (0.5–5 Hz), theta (5–8 Hz), alpha (8–12 Hz), sigma (12–16 Hz) and beta (16–24 Hz).

Even when the SOn process is a gradual phenomenon that unfolds continuously as a function of time (Ogilvie, 2001; Prerau et al., 2014; Siclari et al., 2014), data have to be aligned in order to statistically compare and analyze the process of SOn. Thus, SOn was operationally defined as the first occurrence of an epoch of stage 2 non-rapid eye movement (NREM) sleep. Multivariate autoregressive (MVAR) models, on which the iCOH method relies, were fitted to consecutive 12-s EEG epochs. Outputs were averaged to obtain a single effective connectivity response, representing a 2-min interval per subject, and subsequently averaged across subjects to yield the final group result. Sleep latency (first occurrence of stage 2) was shorter in recovery sleep (4.1 ± 1.2 versus 12.1 ± 1.6 min; SEM; *p* < 0.01; Wilcoxon signed-rank test). Before SOn there was only a single 2-min interval with the contribution of all participants in both conditions. After SOn, 10 min were selected, resulting in five consecutive 2-min intervals, which were averaged to have a unified picture of the connectivity behavior both before and after SOn. The eLORETA version used for the analyses was version 20160611 (most updated at the time of running this study; available as free academic software^1^).

### Isolated Effective Coherence (iCOH)

The algorithm to compute iCOH starts with the estimation of a MVAR model. For details on the mathematical deduction of the iCOH method based on a MVAR model, explained step by step, we refer to (Pascual-Marqui et al., 2014a). Since this effective connectivity analysis is performed at the source level (resulting in spurious results at the scalp level), it is necessary to use a source localization method for the computation of iCOH (Grech et al., 2008; Jatoi et al., 2014). The equation representing iCOH (derived in the frequency domain) takes the following expression:

(1) $$\begin{array}{c} k_{i\leftarrow j}(\omega )=\frac{S_{\varepsilon_{ii}}^{-1}{\left|\overset{⌣}{A}{\left(\omega \right)}_{ij}\right|}^{2}}{S_{\varepsilon_{ii}}^{-1}{\left|\overset{⌣}{A}{\left(\omega \right)}_{ij}\right|}^{2}+S_{\varepsilon_{jj}}^{-1}{\left|\overset{⌣}{A}{\left(\omega \right)}_{jj}\right|}^{2}} \end{array}$$

satisfying the normalization condition: 0 ≤ k_i←j_(ω) ≤ 1 (Pascual-Marqui et al., 2014a). Here, k_i←j_(ω) represents the iCOH value at a given frequency ω between regions of interest (ROIs) *j* and *i*, where the arrow indicates that *j* influences *i* (both indexes range from 1 to *N*, the number of ROIs). The matrix Ă(ω) = I - A(ω), with *I* being the unit matrix of order *N*, relates to the matrix *A*(ω) derived by least square fitting of the MVAR model of order *p* (estimated by the Akaike information criterion). The matrix *S*_𝜀_ represents the covariance of the residual errors of the MVAR model in the frequency domain and improves the result by adding a weight proportional to the accuracy of the accompanying parameters. The software LORETA is able to compute automatically all necessary parameters in this equation (including the optimal order *p* of the MVAR model; *p* = 23 in our case), rendering an iCOH spectrum as output, when providing a given set of EEG data as input.

The particular expression of the iCOH formula (specifically, as seen in the denominator), aims to convey that all connections besides the one of interest (*j* to *i*) are mathematically “severed”. For details about mathematical properties satisfied by iCOH, as well as a comparison with another effective connectivity techniques [the generalized partial directed coherence (gPDC)], we refer to (Pascual-Marqui et al., 2014a,b).

iCOH is normalized and is thus basically independent of the strength of the underlying sources [current source density (CSD); see also (Pascual-Marqui et al., 2014b) for examples of simulated data] similar as it is for coherence and power (Achermann and Borbély, 1998).

### Selection of ROIs

In order to study statistical changes in effective connectivity accompanying the SOn transition, a suitable selection of ROIs needs to be made, aiming to capture the most relevant aspects from a neurobiological perspective, thus, constituting a crucial step in the process of the analysis (Sämann et al., 2011). The inverse solution provided by eLORETA allows for a ROI definition either by providing the Montreal Neurological Institute coordinates of seed regions or by direct selection of Brodmann areas by the user. A total of nine ROIs were selected: the medial prefrontal cortex (MPFC), mid-cingulate gyrus (MCC), posterior cingulate cortex (PCC), bilateral inferior parietal lobule (IPL), bilateral dorsolateral prefrontal cortex (DLPFC) and bilateral hippocampus (H). The rational for these particular ROIs comes from their close relationship with two principal brain networks: the DMN and the CEN (Sporns et al., 2007; Sämann et al., 2011). The first five ROIs define major hubs of the DMN (first two in the anterior, and last three in the posterior part), whereas the DLPFC is the most important hub of the CEN (Andrews-Hanna et al., 2010; Larson-Prior et al., 2011). On the other hand, the hippocampus is sometimes included in the definition of DMN regions, whereas other authors prefer to consider the hippocampus separately as a limbic structure (therefore, excluded from DMN analysis), which nevertheless is acknowledged to interact with cortical hubs of the DMN (e.g., during autobiographical recall) (Buckner et al., 2008; Horovitz et al., 2009; Sämann et al., 2010). Either way, the main reason for adding the hippocampus to our ROI selection is due to its relationship with sleep spindles occurring at the SOn transition, as revealed by intracranial recordings (Nir et al., 2011; Sarasso et al., 2014). Sleep spindles are generated in the thalamus, specifically in the reticular nucleus (Fuentealba and Steriade, 2005; Magnin et al., 2010). Thus, inclusion of the thalamus as a ROI would have made sense. However, we cannot locate thalamic activity with LORETA, as the thalamus is a too deep lying subcortical region for source localization.

The computation of iCOH involves a matrix inversion which limits the number of ROIs that could be included. Eight to nine ROIs (in conjunction with the 127 frequency bins) are the limit avoiding numerical instability in calculating the matrix inversion.

In practice, ROIs were defined by a sphere surrounding the centroid of each ROI. Nevertheless, an unavoidable tradeoff is present, as a small radius would not give the best precision (given that noise effects in the LORETA calculation of spectral current generators do not cancel out when the number of averaged vectors is small), whereas a large radius would tend to produce wrong results for sensitive interactions. In addition, if the radius is too big, the averaged current vector can be even close to 0 in all directions. Thus, as a compromise, the radius for each ROI was defined as 15 mm (Siclari et al., 2014). The cartesian coordinates (Montreal Neurological Institute system, MNI) representing the centroids of our selected ROIs are listed in Table 1.

**Table 1:** Regions of interests (ROIs) selected for the effective connectivity analysis (iCOH) at the sleep onset transition.

Name of region of interest (ROI)	Abbreviation	X (mm)	Y (mm)	Z (mm)
Medial prefrontal cortex	MPFC	0.0	45.0	13.3
Mid-cingulate cortex	MCC	0.0	–17.5	37.5
Posterior cingulate cortex	PCC	0.0	–52.5	27.5
Left inferior parietal lobule	LIPL	–45.0	–45.0	52.5
Right inferior parietal lobule	RIPL	45.0	–45.0	52.5
Left dorsolateral prefrontal cortex	LDLPFC	–37.5	40.0	25.0
Right dorsolateral prefrontal cortex	RDLPFC	37.5	40.0	25.0
Left hippocampus	LH	–21.6	–28.3	–10.0
Right hippocampus	RH	21.6	–28.3	–10.0

*Indicated are the centroid positions of the ROIs in MNI coordinates.*

### Statistical Analyses and Presentation of Results

We carried out statistical tests based on the method of non-parametric randomization of the maximum statistic, which has the advantage of correcting for multiple testing, and of not relying on the assumption of any specific or exact statistical distribution (e.g., Gaussian, Student, Fisher; statistical non-parametric mapping, SnPM) (Nichols and Holmes, 2002; Faes et al., 2012). With SnPM, surrogate permutations (5,000 in our case) are created rendering a histogram which provides the statistical threshold (Pascual-Marqui et al., 2014a).

**FIGURE 1:**
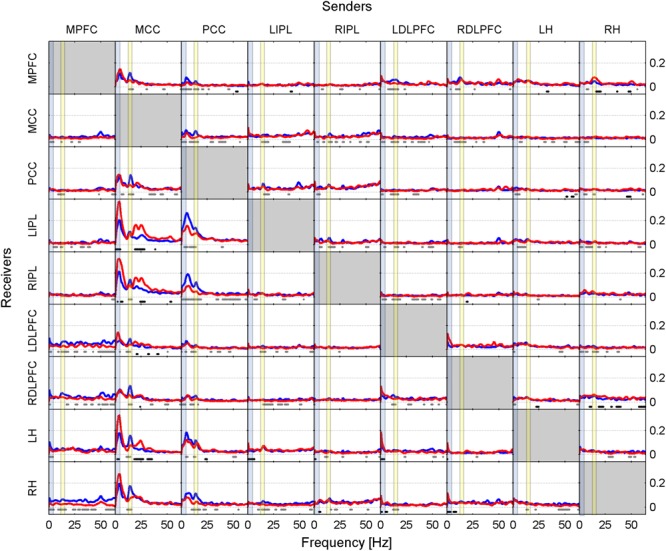
Effective connectivity assessed by iCOH at the SOn transition in baseline. iCOH values representing connectivity strength (effect size) as a function of frequency for any pair of ROIs. Plots arranged as a square matrix of order 9; columns represent “senders”, rows are “receivers” of information flow. For the abbreviations of the ROIs see Table 1. iCOH spectra before SOn (red curves) and after SOn (blue curves) are depicted and the frequency range is from 0.5 to 64 Hz (0.5-Hz resolution). Dots below the curves indicate significant differences between iCOH after SOn vs. iCOH before SOn: gray dots iCOH after SOn > iCOH before SOn; black dots iCOH after SOn < iCOH before SOn. To facilitate orientation, the delta (0.5–5 Hz) and sigma (12–16 Hz) band have been highlighted by light blue and light yellow, respectively.

**FIGURE 2:**
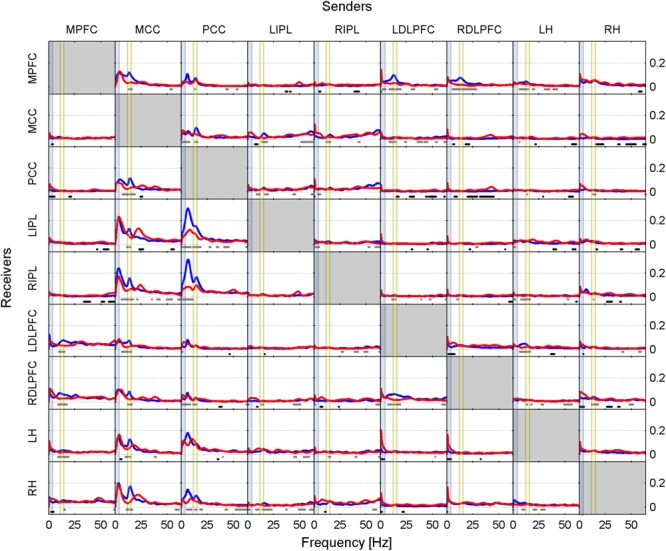
Effective connectivity assessed by iCOH at the SOn transition in recovery after 40 h of sustained wakefulness. For details of the representation see Figure 1.

**FIGURE 3:**
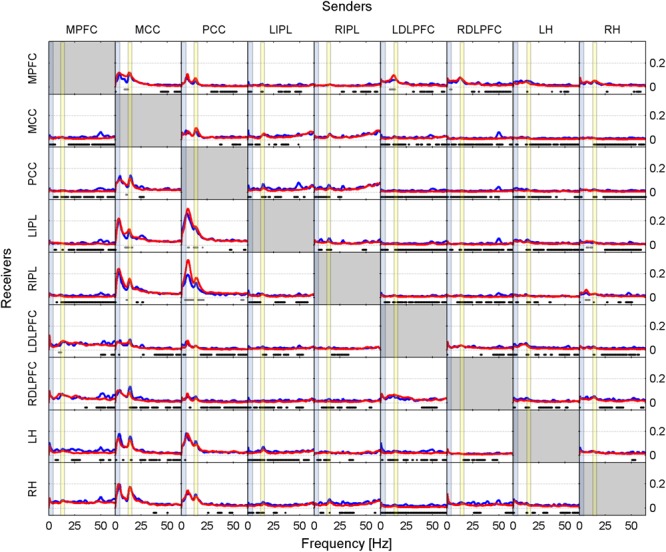
Effective connectivity assessed by iCOH after the SOn transition in baseline (blue curves) and recovery (red curves). For details of the representation see Figure 1. Dots below the curves indicate significant differences between iCOH after SOn in baseline vs. iCOH after SOn in recovery: gray dots iCOH recovery > iCOH baseline; black dots iCOH recovery < iCOH baseline. To facilitate orientation, the delta (0.5–5 Hz)) and sigma (12–16 Hz) band have been highlighted by light blue and light yellow, respectively.

**FIGURE 4:**
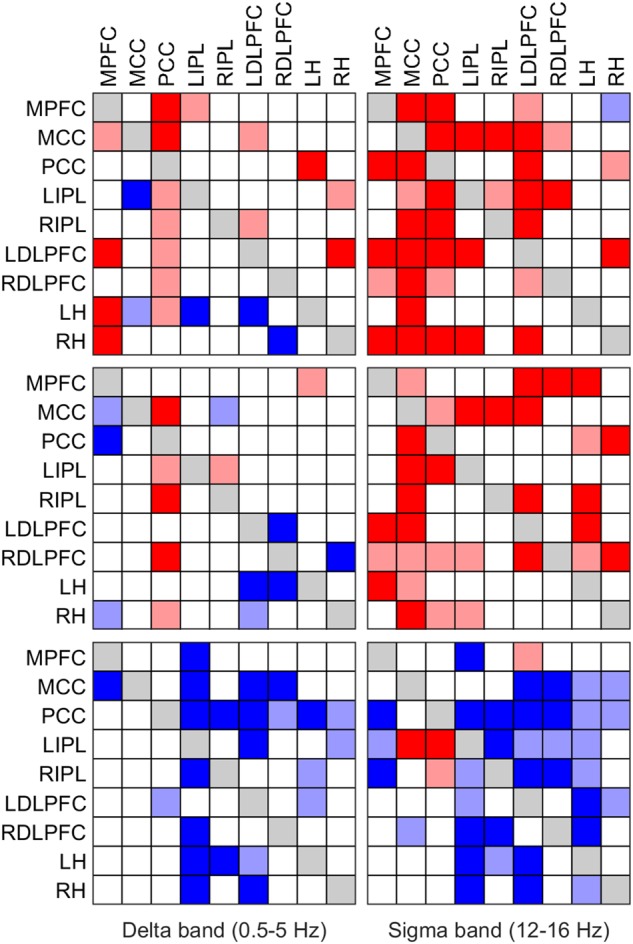
Significant change of iCOH (connectivity) in the delta (left column) and sigma band (right column) at the SOn transition in baseline (top) and recovery (middle) and after SOn between baseline and recovery (bottom). Significant differences between conditions are plotted in matrix fashion; columns represent “senders”, rows are “receivers” of information flow. For the abbreviations of the ROIs see Table 1. Red squares (light and dark) indicate connectivity before SOn < connectivity after SOn (top two rows) and connectivity at baseline < connectivity at recovery (bottom row); blue squares (light and dark) indicate the opposite change. Two significance thresholds were applied to reveal the hierarchy of changes: the lighter colors correspond to *p* < 0.05 (SnPM), the darker ones to *p* < 0.025 (SnPM).

**FIGURE 5:**
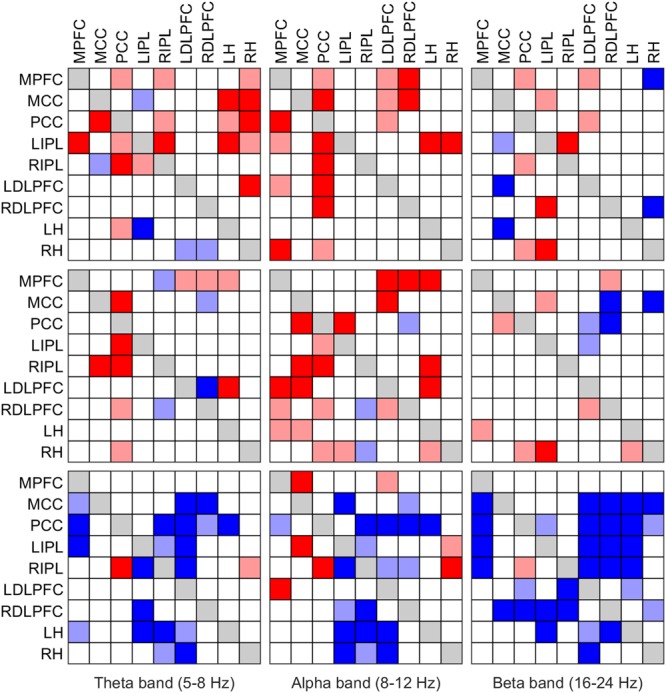
Significant change of iCOH (connectivity) in the theta (left column), alpha (middle column) and beta band (right column) at the SOn transition in baseline (top) and recovery (middle) and after SOn between baseline and recovery (bottom). For details see Figure 4.

Results are presented in two ways in this paper. Firstly, as iCOH values computed by eLORETA, representing connectivity strength (effect size) as a function of frequency for any pair of ROIs (plots are arranged as a square matrix of order 9; Figures 1–3). Columns represent “senders”, while rows are “receivers” of information flow. The correspondence between ROI and position in the square matrix, which is the same for rows and columns, follows the same order as they appear in Table 1. The frequency axis ranges from 0.5 Hz up to the Nyquist frequency (64 Hz), in 0.5 Hz steps. The *y*-axis represents the iCOH values. Dots below the curves indicate differences between conditions A and B (e.g., recovery versus baseline; gray dots A > B; black dots A < B). Secondly, significant differences between conditions A and B are plotted in matrix fashion for specific frequency bands (Figures 4, 5). Red squares indicate A > B, blue ones A < B. All comparisons were corrected for multiple comparisons (see above). Additionally, light and dark red or blue reflect different significance thresholds (light color *p* < 0.05 corrected; dark color approximately *p* < 0.025 corrected) to give an indication on the hierarchy of the changes. We increased the threshold to twice (or if not possible because of being out of range, approximately twice) the 0.05 level.

## Results

### iCOH Spectra

#### Baseline Condition

Figure 1 illustrates the iCOH spectra before and after SOn in the baseline condition [performed on a 2-min window prior to SOn (red curve) and averaged five 2-min windows after SOn (blue curve); group averaged across subjects]. Information flow was clearly asymmetrical with the MCC and PCC as major sources, i.e., drivers and the other regions as receivers, i.e., targets.

Before SOn (Figure 1, red curves), the MCC had the largest impact bilaterally on the IPL and hippocampus with the absolute maximum of iCOH located at 3.5 Hz (delta range) and to a smaller degree to the PCC and bilaterally to the DLPFC. Information flow from the MCC to the MPFC occurred in the theta range (maximum at 5 Hz) and in the beta range (maxima at 20 Hz) bilaterally to the IPL and hippocampus. A further important source was the PCC, with predominantly theta (5.5 Hz) and to a smaller degree sigma (14.5 Hz) flow bilaterally to the IPL and hippocampus. These connections were less strong than those from the MCC.

After SOn (Figure 1, blue curves), one recognizes major changes that occurred after the transition into sleep compared to pre-SOn. Now, the PCC achieved the greatest prominence as a driving source of information flow in the theta range, projecting bilaterally to the IPL [at 5.5 Hz (LIPL) and 6.5 Hz (RIPL)] and hippocampus [at 5.5 Hz (LH) and at 7.5 Hz (RH)]. Further, the PCC projected to all other ROIs in the sigma range (related to spindles) with varying strength. The PCC is anatomically close to the thalamus, the actual generator of sleep spindles (De Gennaro and Ferrara, 2003; Fuentealba and Steriade, 2005), thus, the PCC could be acting as one of its main cortical “relay stations” of spindle propagation (Amico et al., 2014). Secondly, after SOn, the MCC was still exerting influence at various strength levels on all other ROIs mainly in the high delta (4 Hz), low theta (5 Hz) and in the sigma (14.5 Hz) range.

Dots in Figure 1 represent the statistical comparison between the conditions, i.e., the changes that occurred after SOn. Gray dots indicate frequency bins at which effective connectivity was enhanced after SOn, black dots those at which it was decreased. The general picture reveals that prevalent connections present before SOn originating from the MCC were weakened after SOn in the low frequency range (delta and theta bands) and strengthened when originating from the PCC. In addition, causal flow in the beta range (located at 20 Hz and beyond) originating from the MCC was reduced. Finally, synchronization in the sigma band (related to sleep spindles) evolved mainly originating from the PCC and MCC.

Figure 1 also reveals connections from LDLPFC to LH and from RDLPFC to RH very close to 0 Hz. We consider this as a non-biological effect (artifact), i.e., caused by leakage (smearing) of the DC component (0 Hz) also known as Gibbs phenomenon in signal-processing theory.

#### Recovery Condition

Figure 2, similarly to Figure 1, shows iCOH spectra before (red) and after (blue) SOn in recovery after 40 h of sustained wakefulness. As in baseline, information flow was asymmetric with the MCC and PCC as major drivers. The connectivity patterns were like the ones observed in the baseline condition although with a different effect size (see below). Also, the comparison between post- and pre-SOn revealed a similar picture of change as in baseline.

Like in Figures 1, 2 revealed spurious connectivity very close to 0 Hz, e.g., between the left and right DLPFC (Gibbs phenomenon).

#### Statistical Contrast Between Baseline and Recovery

Comparing iCOH spectra prior to SOn between baseline and recovery revealed essentially no significant difference. Figure 3 represents statistical comparison between recovery and baseline sleep for the period after SOn, comparing the blue iCOH spectra of Figures 1, 2.

The overall picture revealed a reduction of iCOH values (effect size) in recovery compared to the baseline following the SOn transition (Figure 3, black dots) across many pairs of ROIs. It affected broad frequency ranges (many of them with low effect sizes), often the beta and gamma range. Increased connectivity due to increased sleep pressure were observed for the following projections: mainly PCC to LIPL and RIPL (theta, alpha and sigma range), and MCC to MPFC, LIPL and RIPL (theta, upper alpha and sigma range). A few pairs of ROIs (out of a total of 72 connections) hardly showed any change: MPFC to RH, PCC to LH, RH to LH, and RIPL to MCC.

### iCOH of Specific Frequency Bands

We also investigated causal information flow in classical frequency bands. Figure 4 illustrates significant effective connectivity changes following SOn in the delta (0.5–5 Hz) and sigma (12–16 Hz) band (left and right columns, respectively) for baseline and recovery (upper and middle rows) and the contrast recovery to baseline (bottom row). Changes in effective connectivity of the theta (5–8 Hz), alpha (8–12 Hz) and beta (16–24 Hz) band are illustrated accordingly in Figure 5. Two levels of the significance (*p* < 0.05 and *p* < 0.025) were applied to provide some information on the hierarchy of the changes, i.e., not all connectivity changes are equally salient in terms of significant increases or decreases as SOn unfolds. This also reduced the type I error. Further, also the strength of the connections and their change revealed by the iCOH spectra (Figures 1–3) helps to get an idea about the hierarchy.

#### Delta Band

In baseline, delta activity (0.5–5 Hz) showed significant changes in information flow between 22 (*p* < 0.05; 11 *p* < 0.025, respectively) pairs of ROIs after SOn, 17 (7) increased and 5 (4) decreased (Figure 4, upper left panel). The PCC acted as a major low frequency synchronizer, connectivity to all ROIs except to the RH was increased (only increases to the MPFC and MCC remained at *p* < 0.025). On the other hand, the MCC, the other major source, showed weakened connections to the LIPL (*p* < 0.025) and the LH (*p* < 0.05). All other changes concerned low effect sizes. Connectivity between the MPFC and the MCC (*p* < 0.05), the LDLPFC (*p* < 0.025), the LH and the RH (*p* < 0.025), between the LIPL and the MPFC (*p* < 0.025), between the LDLPFC and the MCC and the RIPL (both *p* < 0.05), between the LH and the PCC (*p* < 0.025), and the RH and the LIPL (*p* < 0.05) and the LDLPFC (*p* < 0.025) was strengthened. Decreases (weakened connections) occurred from the LDLPFC to the LH (*p* < 0.025), and from the RDLPFC to the RH (*p* < 0.025).

In recovery, information flow between only 16 (8) pairs of ROIs changed [6 (3) pairs less than in baseline], 7 (3) increased and 9 (5) decreased (Figure 4, left middle panel) after SOn in the delta band. The PCC continued to be a main driver of delta flow (however, displaying less significant connections), with increased connectivity strength to the MCC (*p* < 0.025), bilaterally to the IPL, to the RDLPFC (*p* < 0.025) and to the RH (*p* < 0.05). Connections from the MCC to all other ROIs did not change. Weak connections changed as follows: the RIPL showed increased connectivity with the LIPL (*p* < 0.05), and the LH with the MPFC (*p* < 0.05). Decreased connectivity was observed between the MPFC and the MCC (*p* < 0.05), the PCC (*p* < 0.025) and the RH (*p* < 0.05), between the RIPL and the MCC (*p* < 0.05), between the LDLPFC and the LH (*p* < 0.025) and RH (*p* < 0.05), between the RDLPFC and the LDLPFC (*p* < 0.025) and the LH (*p* < 0.025), and between the RH and the RDLPFC (*p* < 0.025).

Comparing baseline and recovery, significant changes in connectivity basically only occurred after SOn. The general picture revealed decreased effective connectivity in the delta band with increased sleep pressure (Figure 4, lower left panel). Twenty-three (16) pairs of ROIs showed decreased iCOH values during recovery compared to baseline. Thus, connectivity strength was higher in baseline. The MCC and PCC as major drivers did not show any change in connection strength with increased sleep pressure except for a reduction in information flow from the PCC to the LDLPFC (*p* < 0.05). All other reductions concerned iCOH values of low effect size.

#### Sigma Band

Sigma activity (power in the sigma band) is closely related to sleep spindles (Dijk et al., 1993). Our results relate to changes in the sigma band. However, for some interpretations (due to the afore-mentioned relationship) we refer to sleep spindles although we did not identify spindles in the present context.

In baseline, 36 (27) significant changes in connectivity occurred, 35 (27) of them representing an increase in the information flow with SOn in the sigma band (12–16 Hz; Figure 4, upper right panel). The MCC occurred to be the main cortical driver of sigma flow in our selection of ROIs, with significantly increased projections to all other ROIs and overall displaying the highest significant increases with the SOn transition (*p* < 0.025). Similarly, the PCC also appeared as an important sigma driver (although less than the MCC), directing connections to all ROIs [*p* < 0.025, except to LIPL (*p* < 0.05)], except to the LH. All other connections showed weak (low effect size) to very weak changes. The MPFC increased transmission to the PCC (*p* < 0.025), bilaterally to the DLPFC and to the RH (*p* < 0.025); the LIPL to the MCC (*p* < 0.025), the LDLPFC (*p* < 0.025) and the RH (*p* < 0.025); the RIPL to the MCC (*p* < 0.025) and the LIPL (*p* < 0.05); the LDLPFC to its right counterpart (*p* < 0.05) and the MPFC (*p* < 0.05), and weakly to the MCC and PCC (*p* < 0.025), bilaterally to the IPL and the RH (*p* < 0.025); the RDLPFC to the MCC (*p* < 0.05) and LIPL (*p* < 0.025). Finally, the RH decreased sigma transmission to the MPFC (*p* < 0.05), but increased it to the PCC (*p* < 0.05) and LDLPFC (*p* < 0.025).

In recovery, 31 (20) connections showed increased information flow between pairs of ROIs [5 (7) less than in baseline] after SOn (Figure 4, middle right panel). Most of them overlapped with those of baseline; some disappeared, and a few weak ones occurred with higher sleep pressure. Again, with the MCC and PCC being the main cortical drivers of sigma flow.

Connectivity in baseline and recovery basically differed only after SOn. Generally, decreased effective connectivity with increased sleep pressure was observed between 35 (20) pairs of ROIs and increased connectivity between 4 (2) pairs (Figure 4, lower right panel). The MCC exerted a stronger impact on the LIPL (*p* < 0.025), and a reduced one on the RDLPFC (*p* < 0.05). The PCC showed heightened information flow to the LIPL (*p* < 0.025) and RIPL (*p* < 0.05). Most other changes concerned weak connectivity.

#### Further Frequency Bands

Changes in effective connectivity of the theta (5–8 Hz), alpha (8–12 Hz) and beta (16–24 Hz) band are illustrated in Figure 5. In the theta and alpha bands at baseline (top row), mostly increased effective connectivity was observed. In the alpha band, the PCC was a major driver of causal flow at the transition. Changes were less obvious in the beta band (fewer connectivity changes with SOn). In recovery (middle row), qualitatively similar changes as in baseline occurred in all bands, although with fewer changes than in baseline. Nonetheless, the PCC could still be identified as the most important ROI causally affecting other ROIs. Comparing baseline and recovery (bottom row), an overall reduction of iCOH occurred after SOn in all frequency bands (in the alpha band, there were several exceptions to this general tendency). If the stricter threshold (*p* < 0.025) was applied, about half of the connections were removed in baseline and recovery (Figure 5, upper and middle row). In the statistical contrast between baseline and recovery (bottom row), most changes remained with stricter threshold (*p* < 0.025), in particular in the theta and beta band.

## Discussion

Overall, we found significant effective connectivity changes, as assessed by iCOH, during the SOn transition, which are congruent with the role of the PCC (a major hub of the DMN) in regulating consciousness (Amico et al., 2014; Herbet et al., 2014). In addition, we showed that, in the cortex, the MCC (and to a lower extent, the PCC) acted as a main relay stations of the thalamus for fast sigma (spindle) synchronization after SOn (Andrillon et al., 2011).

### Relevant Spectral Features of iCOH

#### Baseline

Low frequency transmission arising from the MCC, and to a smaller extent from the PCC to the hippocampus and posterior parts of the DMN appear to be the major causal drivers characterizing the period prior to SOn in the baseline condition (Figure 1, red curves). After the SOn transition the PCC emerged as the most salient structure and principal driver of synchronization, sending theta flow to basically all investigated ROIs (Figure 1, blue curves and Figure 4, top row). The MCC still exerted a prominent role, transmitting causal flow at slightly higher frequencies (high delta/low theta) than before SOn. After SOn, spindle synchronization (sigma band; fast spindle range) occurred throughout all defined ROIs, driven by both the MCC and the PCC. However, this does not necessarily imply that spindles must be considered as a global cortical phenomenon, as local contributions from fronto-parietal areas might be the most important (Andrillon et al., 2011; Del Felice et al., 2014) and additionally may relate to underlying thalamocortical projections (Andrillon et al., 2011).

#### Recovery

Prior to SOn, a delta and theta flow from the MCC (acting as principal source of synchronization), directed to the remaining ROIs was observed (Figure 2, red curves). There was an additional beta flow component from the MCC and PCC, mainly projecting bilaterally to the IPL and hippocampus.

After SOn, firstly theta flow originating from the PCC as main driver, and a secondary delta flow arising from the MCC was present (Figure 2, blue curves). Secondly, sigma synchronization spread across all ROIs (sinks), originating from the MCC and PCC, hence, localizing cortical sources of fast spindles compatible with previous studies (Del Felice et al., 2014; Park et al., 2015). Furthermore, sigma synchronization displayed higher clustering in the frontal lobe during recovery than in baseline, as shown by the enhanced dorsolateral prefrontal contribution (compare Figure 2 with Figure 1; see also Figure 4). The DLPFC, a cortical structure well-known to produce enhanced delta activity during recovery sleep, also received low frequency input, thereby, possibly facilitating the generation of slow waves (De Gennaro et al., 2000; Marzano et al., 2013).

In general, the changes at SOn in recovery were qualitatively similar to the ones in baseline (main differences pertain to the iCOH levels, i.e., effect size), confirming the relevance of the MCC as predominant source of sigma synchronization, with a secondary contribution of the PCC.

#### Recovery Versus Baseline

Most evident was a reduction in connectivity after SOn in recovery compared to baseline (Figure 3, black dots). This effect was most noticeable in the beta range conveying the notion of significantly less beta flow in the sleep deprived brain (associated with cortical and body arousal), although not exclusively restricted to the high frequency range (Kuo et al., 2016; Sena et al., 2016). We can reasonably attribute the significant reduction of strength in cortical communication to the breakdown of effective connectivity accompanying SOn (Massimini et al., 2005; Esser et al., 2009). Nevertheless, there were also connections undergoing a local increase in recovery at certain frequencies. In general, the frequency bands with increased connectivity pertained either the upper alpha or low sigma range, potentially reflecting spindle synchronization.

#### Topographical Properties and Neurobiological Interpretation

In a previous analysis, we estimated the cortical sources underlying brain oscillatory activity at SOn using LORETA (Fernandez Guerrero and Achermann, 2018). The temporal evolution of CSD in different frequency bands was investigated. The most salient findings were observed in the low frequency (delta and theta bands) and in the spindle frequency range (sigma band). Delta activity followed an exponential increase with highest values observed in antero-central areas encompassing also bilaterally the DLPFC, the MPFC and the MCC. Therefore, these regions seem to play a pivotal role at the SO transition, both from an activity and connectivity perspective. Sleep deprivation accelerated the exponential increase and accentuated activity in the afore-mentioned ROIs and eventually in the entire cortex, while effective connectivity decreased after sleep deprivation. Sigma activity followed an inverted U-shape affecting mostly posterior areas, in particular the parietal cortex (the bilateral IPL was also among our ROIs). Sleep deprivation diminished sigma activity levels (including the parietal lobe) and the activity peak shifted to an earlier time (reflecting a fastening of the temporal dynamics) similar to the proposed accelerated temporal dynamics in the DMN connectivity disintegration. Theta and alpha activity increased with time and higher levels were observed with increased sleep pressure while again connectivity was diminished. This diverging response to sleep deprivation of source strength and connectivity in various frequency bands points to the independence of the two measures.

Changes in effective connectivity in the delta band paralleling the SOn transition indicated a greater cortical breakdown affecting fronto-parietal DMN nodes during the recovery condition (Figure 4, left column) (Massimini et al., 2005; Bonhomme et al., 2016). Indeed, integrity of the DMN, particularly the coupling between anterior and posterior parts, has been associated to the degree of conscious arousal or alertness (Horovitz et al., 2009; Chow et al., 2013; Franzen et al., 2013; Britton et al., 2014). For instance, functional connectivity in fMRI studies (based on linear temporal correlations analyses, using DMN nodes as seed regions) applied to the SOn transition have also indicated decoupling of anterior and posterior parts of the DMN with increased sleep depth (Sämann et al., 2011). Consequently, the gradual disruption of anterior to posterior parts of the DMN shown by iCOH might correlate at a subjective level with the progressive fading of consciousness during SOn (Heine et al., 2012; Speth and Speth, 2016).

Our effective connectivity analyses also indicated that this disruptive process occurs at an accelerated rate when the brain is exposed to increased sleep pressure, a behavior also observed with functional connectivity in fMRI (based on cross-correlations, a technique far less rigorous than ours) (Sämann et al., 2010). Thus, Figure 4 would indicate that the reversible “impairment” of consciousness defining the sleep state is more profound during the recovery condition, as a result of reaching a higher network disconnection than in baseline (Boly et al., 2008; Barrett et al., 2012). Hence, the fronto-parietal disconnection (Figure 4, left middle panel) may be seen as a temporally accelerated connectivity change reaching a deeper loss of conscious awareness.

Effective connectivity analyses pointed to the PCC as the major hub affecting effective connectivity breakdown during SOn. The predominance of the PCC holds in terms of both the largest effect size and in the frequency of statistically significant differences observed as critical sending hub in the delta, alpha and sigma frequency bands (also for theta when the more lenient threshold is considered). Indeed, this structure has been shown in many studies to play a crucial, necessary, albeit possibly not sufficient, role in maintenance of a normal state of consciousness, or alternation between different states of consciousness (Vogt and Laureys, 2005; Amico et al., 2014; Herbet et al., 2014; Leech and Sharp, 2014). Moreover, the PCC plays a crucial role in the normal DMN functioning, and its relevance is highlighted by the fact that it has the highest metabolic rate of the brain and the highest degree of functional connectivity to other brain areas in resting-state analyses performed with fMRI studies (Vogt and Laureys, 2005; Sämann et al., 2011).

Finally, as another prominent feature the hippocampus received less delta flow from the prefrontal cortex in the recovery condition, and in turn, sent less flow itself (Figure 4). The hippocampus is a structure known to be involved in both short-term and long-term memory, and has also been implicated in working memory in conjunction with the prefrontal cortex (Lavenex and Amaral, 2000; Leszczynski, 2011). Furthermore, the hippocampus is documented to play a pivotal role in transferring of declarative memories gathered during the day to the cortex for long-term storage, a process that happens principally during stage 2 and deep sleep (Born et al., 2006; Rasch and Born, 2013). We hypothesize that this prefrontal-hippocampal effective connectivity breakdown may constitute a neurobiological explanation for impaired declarative memory consolidation under conditions of sleep deprivation (Lavenex and Amaral, 2000; Gais and Born, 2004).

In general, the topology of the network indicating major statistical changes unfolding with the SOn transition in the sigma range (Figure 4, right column) exhibited a great similarity between the two conditions, although the number of statistically significant connections was a bit higher at baseline (36 significant connections, compared to 30 in the recovery condition). The lower number of significant connections in the recovery condition could indicate a reduced capacity to generate and propagate spindles throughout the cortical mantle when subjects are under higher sleep pressure (Olbrich et al., 2014). This may also relate to the inverse relationship between delta and sigma activity (delta activity increasing with sleep pressure), and as such leading to lower sigma activity (less spindles) in recovery sleep (Carrier et al., 2001; Finelli et al., 2001b). The topology was mainly governed by fronto-parietal connections, generally indicating a statistical increase in iCOH values, hence, in agreement with sigma synchronization at SOn (De Gennaro et al., 2000; Anderer et al., 2001).

As we defined the sigma band as 12–16 Hz, effective connectivity results relate to fast (rather than slow) spindles (Aeschbach and Borbély, 1993; Aeschbach et al., 1997; De Gennaro and Ferrara, 2003). Although spindles have a thalamic origin (LORETA cannot localize deep subcortical structures), as a relay, the major cortical hub acting as a driver for spindle synchronization (in this case, fast spindles) was the MCC, both in baseline and recovery conditions (Amico et al., 2014). Given that fast spindles show maximum power spectral density along the parietal lobe, the spatial location of MCC is optimal for being the driver of fast spindles (Anderer et al., 2001; Marzano et al., 2013). This structure is compatible with other studies of source localization using LORETA (Anderer et al., 2001; Andrillon et al., 2011; Del Felice et al., 2014).

For both delta and sigma activity, the general trend, with few exceptions, was a reduction of connectivity strength in the recovery condition. The spatial organization of the network indicating significant changes remains remarkably similar in the two conditions, suggesting that the recovery condition is not characterized by a new spatial configuration, different from baseline, but rather by a loss of connectivity strength within the same main fronto-parietal networks. On the other hand, the increases of connectivity with the transition may reflect an endogenous mechanism to reinforce the gradual disengagement from the external environment (e.g., by enhancing slow wave activity spreading, with a prominent role of the IPL) or, quite the opposite, counter-balancing mechanisms that are impeding the consolidation of the SOn process (as, e.g., in insomnia patients). If the reinforcement hypothesis is correct, healthy sleepers should exhibit increased connectivity to a greater extent (either in effect size, frequency of significance or both) than insomniacs. On the other hand, if increased connectivity is due to a counter-balancing mechanism hindering SOn, it should be more noticeable in insomniac than healthy subjects.

The anterior-posterior decoupling following the SOn transition has also been observed using yet another effective connectivity technique different from the iCOH, the Direct Transfer Function (DTF) (De Gennaro et al., 2004). In this regard, the DTF showed, for the period preceding SOn (emergence of first spindle or K complex), a prevalence of occipital to frontal information flow in the delta, theta and alpha bands. However, after SOn, the directionality pattern inverted, and the predominant direction of transmission was fronto-parietal to occipital at all frequency bands. Although this analysis was based on the scalp EEG and did not define brain networks, the observed behavior was compatible with a breakdown of the DMN. In addition, a later study confirmed and extended the previous finding based on the DTF (De Gennaro et al., 2005). In this study, the effects on connectivity resulting from total sleep deprivation were also assessed by means of the DTF. With increased sleep pressure, the anterior-to-posterior directionality of coupling could be already detected before the SOn transition, therefore, constituting a time advance shift compared to the baseline condition. This speed-up of the dynamics has also been observed investigating the temporal evolution of the cortical sources of oscillatory activity at the SOn transition (Fernandez Guerrero and Achermann, 2018). Regarding the above-mentioned mentioned DTF connectivity patterns preceding SOn, our results do not necessarily contradict these findings given that we used different time intervals and pursued a different approach to investigate connectivity (iCOH instead of the DTF). De Gennaro et al. (2005) defined the “pre-sleep onset period” as the interval of 5 min before the first occurrence of a spindle or K complex (first epoch of stage 2), compared to the “post-sleep onset period”, of 5 min duration. Therefore, our analyses intervals differ considerably as we analyzed 2 min prior to and 10 min after SOn. We did not observe significant differences between baseline and recovery in the 2-min interval preceding SOn. Nonetheless, we cannot exclude that effects would have been detected with longer intervals. However, our participants fell asleep very fast prohibiting the analysis of a longer pre-sleep interval. Furthermore, variability in the 2-min intervals prior to SOn might have been too large, i.e., power of our study might have been insufficient given the relatively small number of participants (see section “Limitations”).

Our connectivity analyses revealed to some degree interhemispheric asymmetries, e.g., between the LDLPFC and the RDLPFC or the LH and the RH in the delta and sigma range. Interhemispheric asymmetries during SOn have also been reported at the level of power spectral density (Ioannides et al., 2017). With our small sample size, we are not confident to make strong claims, but the asymmetries might be related to functional laterality (participants were right-handed).

Finally, a fRMI study and an EEG based one of functional connectivity and graph theory also concluded that the SOn transition is accompanied by a breakdown of cortico-cortical connectivity as sleep progresses into slow wave sleep (Spoormaker et al., 2010; Vecchio et al., 2017). The delta and theta band revealed lower levels of small-world properties, indicative of reduced connectivity after SOn, while the opposite was observed for sigma activity reflecting emerging spindle synchronization (Vecchio et al., 2017). The breakdown in cortico-cortical connectivity between anterior and posterior nodes of the DMN serves as a neurophysiological explanation for disengagement from the external world, as it hinders the capacity of cortical areas to integrate information received from other brain areas. However, before entering into slow wave sleep, cortico-cortical connectivity was observed to be higher than in wakefulness, which fits with our data (in particular, synchronization of spindles). Additionally, sleep studies also using graph theory in order to measure small-world properties are consistent with the network reorganization we observed after SOn (Ferri et al., 2007, 2008).

#### Limitations

Some limitations of the current analysis are important to note. Twenty-seven EEG electrodes were used which is at the lower limit for source localization and a general head model was employed. We cannot neglect the blurring of the solutions that increases for deeper sources (Grech et al., 2008; Pascual-Marqui et al., 2014a). The blurring introduced by the LORETA method may affect not only the current density maps, but also connectivity results (Jatoi et al., 2014).

A new method has been proposed recently called “innovations orthogonalization” in order to tackle some arising pitfalls in the application of iCOH, due to leakage or mixing of signals produced by both volume conduction and the low spatial resolution of techniques such as LORETA (Pascual-Marqui et al., 2017). Through this type of correction, spectral responses are more accurate; however, we reckon that the subtle correction performed by the innovations orthogonalization method does not conflict with our results, as it mostly pertains a subtle spectral resolution of effective connectivity.

Also, our selection of ROIs has an impact on the results and their interpretation. As we, e.g., did not differentiate between hemispheres for MPFC, MCC and PCC or between dorsal or ventral MPFC and dorsal or ventral hippocampus, interpretations may be limited. However, we were cautious with the selection of ROIs (see section “Materials and Methods”) and think that the global picture was captured with our selection.

Spindles in the hippocampus were observed in intracranial recordings of epileptic patients (Sarasso et al., 2014). Given that the precision of LORETA is expected to decrease with increasing depth, we do not know whether spindles occurred in the hippocampus. Nonetheless, spectral changes in the sigma range of iCOH passed the statistical tests, so we may assume that they relate to hippocampal activity.

Further, a homogenous sample of eight healthy good sleepers was included in the analyses which is a relatively small sample. However, changes congruent to published findings were observed but we cannot rule out that statistical power was insufficient.

Finally, we cannot exclude that some of the connectivity patterns observed after SOn resemble NREM sleep in general. However, the comparison pre-sleep to post-sleep is specific for the SOn process.

## Conclusion

iCOH proved to be a valuable tool to reveal the effective connectivity patterns at the transition into sleep revealing the spectral characteristics of the information transmission. A posterior to anterior decoupling of the DMN in the low frequency range was observed, reflecting the progressive disengagement from the external environment at the transition to sleep. The PCC played a major role in the unfolding of the SOn transition, guiding the other nodes, particularly, in the delta-theta range. Furthermore, the MCC appeared as a principal cortical relay station of the thalamus in sigma synchronization (related to spindles) throughout the cortex. Lower overall cortical connectivity was present after sustained wakefulness, thus, the SOn transition exhibited a smaller connectivity reduction than in baseline but still leading to a disconnection of the major nodes of the DMN.

## Ethics Statement

The local ethical committee for research on human subjects approved the study protocol.

## Author Contributions

AFG and PA designed the analyses and wrote the paper. AFG conducted the analyses.

## Conflict of Interest Statement

The authors declare that the research was conducted in the absence of any commercial or financial relationships that could be construed as a potential conflict of interest.

## Footnotes

1 http://uzh.ch/keyinst/loreta
